# A Wireless Pressure Sensor Integrated with a Biodegradable Polymer Stent for Biomedical Applications

**DOI:** 10.3390/s16060809

**Published:** 2016-06-02

**Authors:** Jongsung Park, Ji-Kwan Kim, Swati J. Patil, Jun-Kyu Park, SuA Park, Dong-Weon Lee

**Affiliations:** 1 MEMS and Nanotechnology Laboratory, Mechanical Engineering, Chonnam National University, Gwangju 61186, Korea; atoz153@gmail.com (J.P.); swatipatil39@gmail.com (S.J.P.); 2 Department of Defense Science and Technology, Gwangju University, Gwangju 61743, Korea; jkkim79@gwangju.ac.kr; 3 CGBio Co. Ltd., Jangseong-gun 57248, Korea; pjk23@naver.com; 4 Department of Nature-Inspired Nanoconvergence Systems, Nanoconvergence Mechanical System Division, Korea Institute of Machinery and Materials (KIMM), Daejeon 34103, Korea; psa@kimm.re.kr

**Keywords:** pressure sensor, LC circuit, SU-8, wireless sensing, polymer stent

## Abstract

This paper describes the fabrication and characterization of a wireless pressure sensor for smart stent applications. The micromachined pressure sensor has an area of 3.13 × 3.16 mm^2^ and is fabricated with a photosensitive SU-8 polymer. The wireless pressure sensor comprises a resonant circuit and can be used without the use of an internal power source. The capacitance variations caused by changes in the intravascular pressure shift the resonance frequency of the sensor. This change can be detected using an external antenna, thus enabling the measurement of the pressure changes inside a tube with a simple external circuit. The wireless pressure sensor is capable of measuring pressure from 0 mmHg to 230 mmHg, with a sensitivity of 0.043 MHz/mmHg. The biocompatibility of the pressure sensor was evaluated using cardiac cells isolated from neonatal rat ventricular myocytes. After inserting a metal stent integrated with the pressure sensor into a cardiovascular vessel of an animal, medical systems such as X-ray were employed to consistently monitor the condition of the blood vessel. No abnormality was found in the animal blood vessel for approximately one month. Furthermore, a biodegradable polymer (polycaprolactone) stent was fabricated with a 3D printer. The polymer stent exhibits better sensitivity degradation of the pressure sensor compared to the metal stent.

## 1. Introduction

The microelectromechanical system (MEMS) technology is capable of high levels of integration and miniaturization and is adequate for thin-film production. Leveraging these characteristics, many biotechnological (or medical implant related) applications became possible; this led to recent studies regarding implantable or wearable devices [1,2,3,4,5]. The implantable sensor technology can be broadly divided into active and passive devices. Active devices possess high levels of functionality, because the electronic circuitry and power supply devices are integrated together. However, its complexity, various packaging methods, and the lack of reliability of their linkage with various devices are still drawbacks. Hence, such kinds of devices are unsuitable for implantation in the human body or in the blood vessel because the integrated power supply devices have associated lifetime constraints. On the other hand, passive devices are integrated with an inductor coil to wirelessly obtain the electric power required for sensor operation and have very simple structures. For example, integrating the inductor coil with a capacitive sensor or a surface acoustic wave sensor, passive devices can be used for various functions, such as the detection of pressure, temperature, and the presence of ions [6]. Passive sensors are powered through the mutual inductance between an external coil and a sensor coil. This method is suitable for implantable micro-devices and can generally be applied to overcome the limits of integrated battery power supplies. The passive inductively coupled devices are mostly used in wireless (radio) system with better performance, due to the better specifications and good performances over a wide frequency band [7]. Recently, various researchers have been focused on the development of implanted devices that are low-powered, highly efficient, and reliable and have a high data rate [8].

Chen *et al.* [1] fabricated a pressure sensor based on polyimide and evaluated its performance. The sensor, integrated with an inductor-capacitor (LC) circuit, is capable of being wirelessly powered; the changes in pressure are measured by evaluating capacitance changes. Because the sensor is envisaged for miniaturization and high pressure-sensitivity, it was designed to use a frequency band in the GHz region. Its high resonance frequency results from its highly miniaturized sensor size but can lead to serious problems such as cell disruption when implanted in the human body [9]. Gong *et al.* [2] fabricated another pressure sensor with higher sensitivity, using gold nanowires. This sensor reduced the power consumption during the pressure measurement by minimizing the current flow and suggests the possibility of new electrode materials. However, the power of this sensor was supplied through electric wires, which limits its application to wearable elements for which mobility is essential. Zang *et al.* [3], Schwartz *et al.* [4], and Michael *et al.* [5] also suggested other types of sensors with various levels of flexibility, low power, *etc.* and noted the existence of limits regarding biocompatibility, signal interference, and wireless power supply. 

The main purpose of this research is to fabricate a wireless pressure sensor comprising an LC circuit integrated with a biodegradable polymer stent as a new medical device. The micromachining-based inductor-capacitor circuit has a simple design and can be fabricated in sizes below a few millimeters. The wireless pressure sensor body is fabricated with a photosensitive SU-8 polymer, and a resonant circuit is integrated with the sensor. The post-treated SU-8 polymer has excellent biocompatibility and does not require additional packaging processes [10,11]. Moreover, the polymer sensor can be mass-produced because it is suitable for existing semiconductor processes. The pressure sensor is designed with two passive elements, which can be wirelessly supplied with power and typically operate at frequencies in the range of hundreds of kHz to a few MHz [12]. The pressure sensor has a surface area of approximately 3 × 3 mm^2^ and a thickness of 150 µm and is capable of wirelessly monitoring the surrounding pressure. The polymer stent, fabricated by using a biodegradable polymer (polycaprolactone) with a 3D printer, does not affect radio frequency (RF) signals during measurements, unlike metal stents. Moreover, complex processes such as laser processing are not required during the stent fabrication, so fabrication of complex structures can be done more easily. By integrating the stent with a mobile measuring instrument, a patient will be capable of self-monitoring the re-adsorption inside human blood vessels in real time, thereby minimizing the need for exposure to radiation (including X-rays) and thus preventing the patient exposition to additional health-related risks.

## 2. Theory and Design

**Figure 1:**
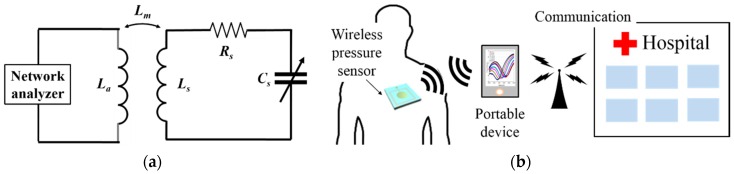
Schematic of (**a**) wireless and battery-less inductive-capacitive (LC) resonant circuit; (**b**) wireless blood pressure monitoring using the LC resonant sensor combined with a polymer stent.

The wireless pressure sensor proposed in this paper is based on inductive coupling. The pressure sensor is powered without the need for an integrated power supply, and the change in pressure can be wirelessly detected by the change in the capacitance connected to the inductor. The sensor comprises an inductor coil and a metal-insulator-metal (MIM) capacitor. Figure 1a presents a schematic circuit diagram of the sensor and output measuring system. Through an impedance measuring instrument connected to an external antenna, the characteristics of the sensor resonance frequency can be measured. Linking this information with external detection and processing systems, additional types of application can become possible later on, as shown in Figure 1b. The pressure sensor proposed in this paper is integrated with a biodegradable polymer stent; it has an area of approximately 3 mm^2^ and operates at around 200 MHz. The energy loss due to RF signals can be affected by numerous variables, including the resonance frequencies between the two coils and their orientation. The loss of energy is related to the interaction of environment with biological tissue and which depends on frequency selection. If the thickness of the tissue between two coils is less than one-tenth of the wavelength of the RF signal, then the energy absorption in the tissue is proportional to the square of the signal frequency. Therefore, the operational frequency of the system cannot be too high [9]. In other words, a sensor with a high resonance frequency is capable of delivering high energy, but the sensor’s resonance frequency must be lowered because of the increase in energy absorbed by the human body that such a high operating frequency would imply. On the other hand, high quality factor values are difficult to obtain with low resonance frequencies. Therefore, the resonance frequency should be appropriately selected in accordance with the environmental factors of the particular application. In this study, the sensitivity is set to be as high as possible in the pressure range of 0 mmHg to 230 mmHg. The sensor’s resonance frequency can be simply expressed as a function of its inductance (*Ls*) and capacitance (*Cs*), as shown in Equation (1) [13].

(1) $$\omega_{0}=2\pi f_{0}=\frac{1}{\sqrt{L_{s}C_{s}}}$$

**Figure 2:**
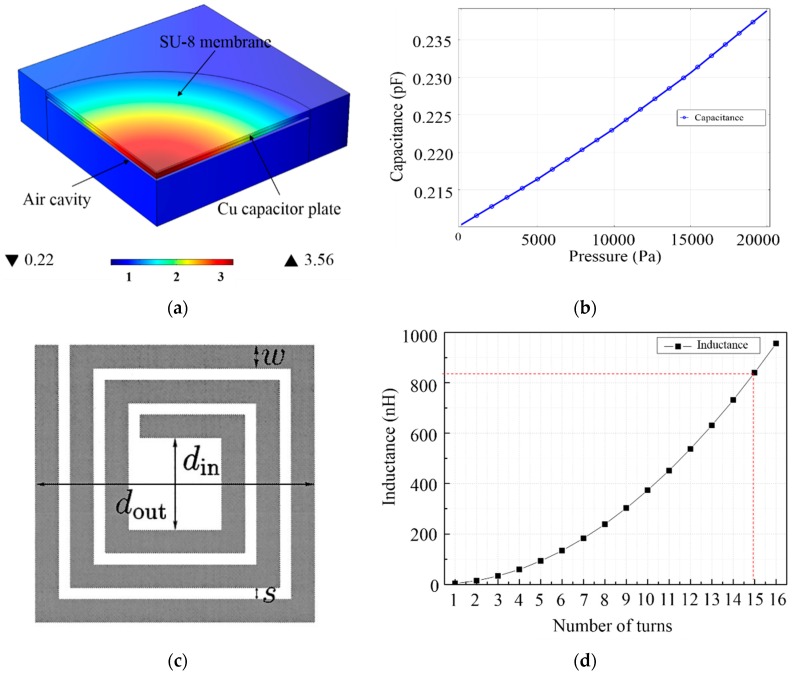
(**a**) Finite element analysis of the displacement; (**b**) capacitance variation as a function of pressure; (**c**) square inductor coil calculation factors; (**d**) inductance variation as a function of the number of turns in the coil.

The inductance value of the inductor remains constant after fabrication; the capacitance of the capacitor is variable with change in the applied pressure. The change in resonance frequency can be estimated by Equation (1) based on the capacitance variation caused by the change in pressure. The MIM capacitor has a 1-mm diameter, and the intermediate insulation layer is made of either polydimethylsiloxane or a closed air layer. A finite element analysis software program is used to analyze the change in capacitance in the 0–150-mmHg pressure range—which is the operating range of the wireless pressure sensor at normal blood pressure condition. To facilitate the analysis, only one quarter of the capacitor was modeled, with a maximum value of diaphragm displacement of approximately 3.56 µm, as shown in Figure 2a. When the pressure increases from 0 mmHg to 150 mmHg, the pressure sensor shows an increase in capacitance up to a maximum value of approximately 0.235 pF, as shown in Figure 2b. Thus, the inductor was designed with the appropriate value of inductance that is needed to obtain a resonance frequency of 200 MHz in the wireless pressure sensor. The integrated inductor coil supplies the electric power required inside the sensor using the electromagnetic field generated by the external antenna. A bigger inductor coil collects more power for the sensor and *vice versa*. In this study, both the width and thickness of the inductor coil are set at 15 µm, considering the process variations. Altering the number of turns of the inductor coil, the desired degree of miniaturization can be achieved. The inductance (Ls) obtained for direct current can be calculated using Equation (2) [14].

(2) $$L_{s}=\frac{\mu n^{2}d_{\text{avg}}}{2}\left[\ln\left(\frac{2.07}{\rho_{s}}\right)+0.13\rho_{s}^{2}\right]$$

where µ is the permeability of vacuum, *d*_avg_ = 0.5(*d*_out_ + *d*_in_), *ρ* = (*d*_out_ − *d*_in_)/(*d*_out_ + *d*_in_), and n is the number of turns for inductor coils (see Figure 2c). The size of the pressure sensor is limited to 3 × 3 mm^2^ because of the need to integrate with the polymer stent. Figure 2d shows the inductance values achievable with the surface area (number of turns). Considering the miniaturization requirements for the pressure sensor, the inductor coil with 15 turns was set and resulted in an inductance value of approximately 839 nH. The resonance frequency of a wireless pressure sensor, based on the series connection of inductor (15 turns) and the previously discussed capacitor, is of approximately 200 MHz.

## 3. Fabrication of the LC Resonant Sensors and the Stents

**Figure 3:**
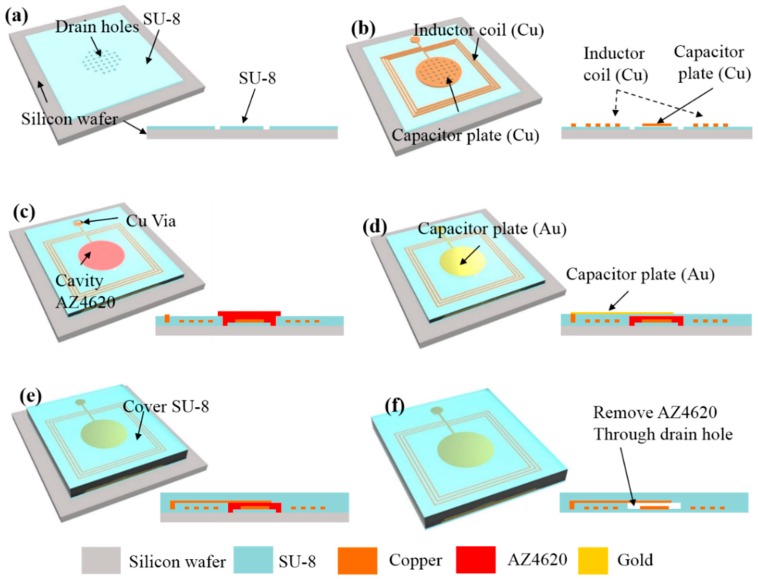
(**a**–**f**) A process flow for the fabrication of wireless pressure sensors: (**a**) patterning of SU-8 photoresist; (**b**) electroplating for coils and a bottom electrode on the SU-8 layer; (**c**) patterning of AZ 4620 sacrificial layer and electroplating for copper interconnection; (**d**) Au deposition and patterning for a top electrode; (**e**) SU-8 coating on the LC circuits; (**f**) removal of sacrificial layer.

An LC resonant wireless pressure sensor was fabricated by MEMS system processes, based on a photosensitive and biocompatible SU-8 polymer. Using MEMS processes, the miniaturization and mass production of a sensor is possible, thereby enabling a batch fabrication of approximately 177 pressure sensors on a 100-mm Si wafer. To improve the pressure sensor’s reliability, an inductor and capacitor were made of Gold (Au) and Copper (Cu), which have excellent electrical conductivity. A total of seven photomasks were used for the fabrication of the pressure sensors. The stepwise process flow for the fabrication of wireless pressure sensor is illustrated in Figure 3. The pressure sensor comprises a capacitor with a 1-mm diameter of a circular parallel plane plate inside a rectangular inductor coil with 15 turns, composing an LC-resonant circuit.

As shown in Figure 3a, an oxide film with a thickness of 500 nm as a sacrificial layer was grown on the surface of the 100-mm silicon wafer. The spin-coating of SU-8 2007, a sensor body with a thickness of 7 µm, was fabricated by a photolithography process. A porous layer with 10-µm diameter holes was formed in the center of the sensor to facilitate the removal of the AZ4620 resist and form the capacitor cavity. In the second step, an appropriate pattern was drawn by a lift-off process using a GXR 601 46cp photoresist, as seen in Figure 3b.

Using an e-beam evaporator, Titanium (Ti) and Gold (Au) layers were deposited with 10-nm and 100-nm thicknesses, respectively, and formed a photoresist mold (AZ4620) with a thickness of 20 µm. Then, an electroplating process was performed to deposit a thick layer of Cu with a thickness of 15 µm on the previously patterned Ti/Au layer. After that, SU-8 was spin-coated on top of the inductor, and this polymer layer electrically isolated the inductor coil during operation in a liquid condition. In the next step, a 30-µm-thick photoresist was spin-coated and patterned on the top surface, which acted as a sacrificial layer to form a cavity between the two capacitor plates. Then, to reduce the reflow of the photoresist, photoresist patterns were hard-baked at 90 °C for 10 h.

In the third step, as shown in Figure 3c-d, to create good electrode contact between the lower and upper capacitor plates, the electroplating process was repeated and after that fabricate the upper capacitor electrode. The upper capacitor electrode was deposited with Ti and Au with 10-nm and 100-nm thicknesses, respectively, and then completed with metal patterning, as shown in Figure 3d. An air tightness and protection of the metal layer, a SU-8 pattern with a thickness of 150 µm was formed on the fabricated device, as shown in Figure 3e.

**Figure 4:**
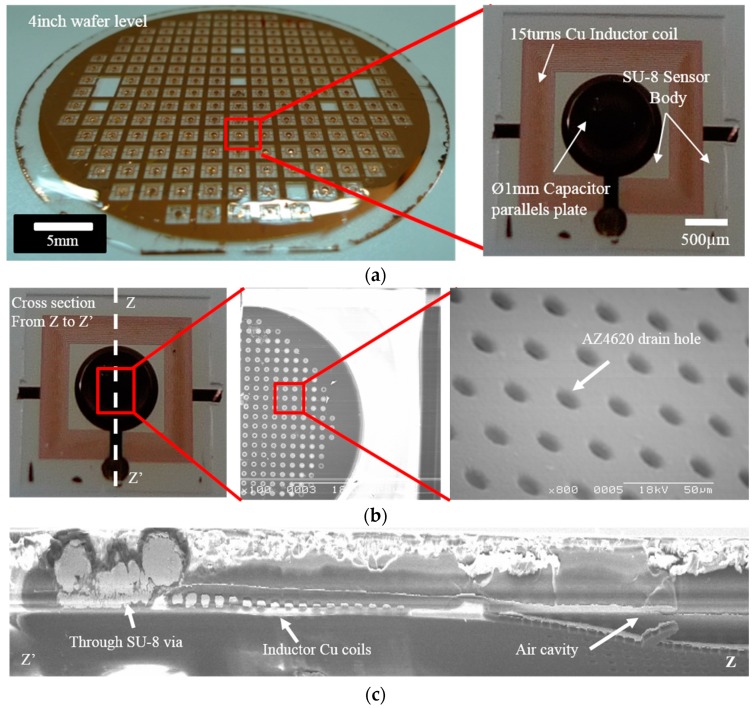
(**a**) The actual photo images; (**b**) SEM images; and (**c**) cross-sectional view of wireless pressure sensor.

Finally, the sensor elements were separated from the silicon wafer by etching the oxide film, and the AZ4620 resist was completely removed from the capacitor plates by acetone solvent. Figure 3f shows the fabricated wireless pressure sensors available for testing. The wireless pressure sensors, with thicknesses of 150 µm and with lengths and widths of 3.16 mm and 3.13 mm, respectively, were fabricated on the 100-mm silicon wafer. Figure 4a shows the actual photographs of the fabricated wireless pressure sensor. The drain holes formed on the first SU-8 layer, as seen in Figure 4b, which facilitated the removal of the photoresist used for the sacrificial layer. The cross-sectional SEM image of the pressure sensor is displayed in Figure 4c.

**Figure 5:**
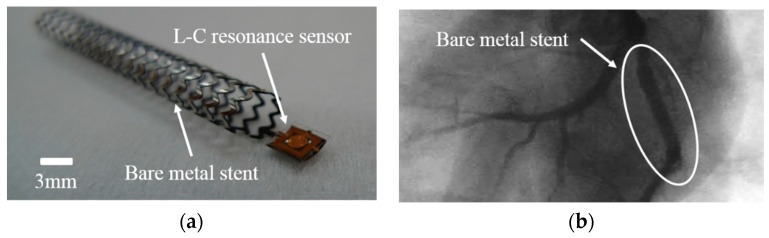
(**a**) Optical image of bare metal stent integrated with the wireless pressure sensor; and (**b**) X-ray image of animal test for the biocompatibility of the pressure sensor.

The stent inserted into the cardiovascular blood vessel is made of metal and is generally fabricated by laser processing. Figure 5a shows an optical image of a homemade bare metal stent made of Cobalt-Chromium (Co–Cr) material, fabricated for integration with the pressure sensor developed in this study. To verify the biocompatibility of the pressure sensor, a stent implantation was performed inside the cardiovascular blood vessel of a pig after the integration of the metal stent and sensor. No specific phenomena caused by the pressure sensor were observed inside the blood vessel for approximately one month. The X-ray image of the animal test for the biocompatibility of the pressure sensor with the integrated metal stent can be seen in Figure 5b. However, the metal-based structure interfered with a resonant circuit operation and was ultimately confirmed as unsuitable for application as a biodegradable stent. Therefore, in this study, to obtain biodegradability and minimize the signal interference with the pressure sensor, a polymer stent was fabricated using a 3D printer. Polycaprolactoneis was used as polymer stent because it is a biodegradable polymer [15] and can be shaped in the form of a conduit with a 5-mm diameter. The biodegradable polymer stent is capable of minimizing angiostenosis and neointimal growth. Unlike the metal stent, the biodegradable polymer can also reduce the costs of reoperation caused by severe stenosis inside the blood vessel due to its biodegradability in the body. Considering these characteristics, biodegradable polymer stents are expected to become the new-generation stents. Moreover, traditional metal stents are fabricated by processing microtubules; therefore, it is difficult to form complex three-dimensional structures, and they present rough surfaces due to the heat generated by the laser processing. Therefore, an additional process is necessary for physical and chemical smoothing of the surface; this can lead to the metal stent deformation. The polymer stent fabricated by a 3D printer, on the other hand, is directly and accurately shaped by the spray nozzle of the printer. No post-treatment process is required, and the stent can be easily processed and fabricated into various shapes and forms using various materials on a sub-mm scale. Manu *et al.* [16] designed a cyborg ear with device dimensions measured in cm^2^ with a 3D printing method. Jacob *et al.* [17] fabricated 3D electrically small antennas by conformal printing of metallic inks with dimensions of 1 cm^2^ and a metal width of 100 µm [17]. Our fabricated MEMS-based sensor has dimensions of approximately 3 mm^2^ and a metal width of 20 µm, whereas 3D-printed metal sensors have dimensions of 1 cm^2^. Therefore, considering the size of fabricated device, the MEMS-based sensors have a smaller size. Therefore, the smaller sensor size can easily integrate with the polymer stent to miniaturize the device.

## 4. Results and Discussion

The yield of the pressure sensor separated from the 4-inch wafer was roughly above 50%. The 170-μm-thick miniaturized LC wireless pressure sensor was integrated with the polymer stent using a biodegradable epoxy resin. The cross section of the sensor was observed by scanning electron microscopy, which clearly shows the MIM and inductor coil components of the sensor (Figure 4c). The existence of copper via electrically connected with the upper and lower capacitor plates could be confirmed. The gap of the MIM capacitor insulator is approximately 30 µm. Finally, the drain hole can be ultimately sealed using an SU-8 photoresist. Although the inductor coil of the sensor is 15-µm-thick, the thickness decreased toward the center due to the limits of electroplating. This caused a difference in the sensor’s resonance frequency, which depends on the sensor position within the surface of the 4-inch Si wafer. The resonance frequency of the pressure sensors were measured in a range from 170 MHz to 210 MHz. A variable frequency was observed due to the uneven electric current density during the electroplating process but these problems can be resolved by electroless plating or by the optimization of these processes.

**Figure 6:**
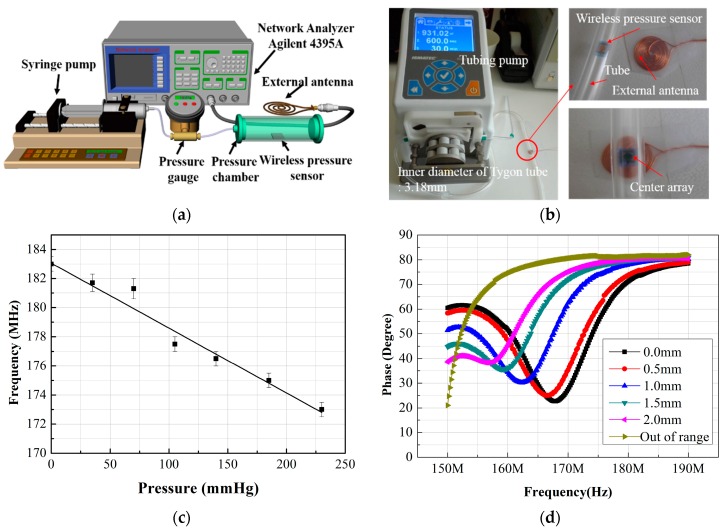
(**a**) Schematic of the experimental setup; (**b**) photographs of a fabricated sensor placed in a pressure-controllable tube; (**c**) resonance frequency variation as a function of the applied pressure; (**d**) phase as a function of frequency with different working distance between the sensor and the external antenna.

A homemade experimental setup was used to evaluate the resonance frequency change with the pressure sustained by the sensor, as shown in Figure 6a. The phase change and resonance frequencies were measured by an impedance analyzer (Agilent 4395A) connected to the external antenna. The wireless pressure sensor was fixed to a specially designed pressure chamber, and the pressure in the chamber varied from 0 to 230 mmHg in 35-mmHg intervals (see Figure 6b). This value was chosen because of the general criteria for determining arteriosclerosis or angiostenosisis based on differential blood pressures of 35 mmHg between the peak blood pressures. In the present experiment, the resonance frequency of the pressure sensor at atmospheric pressure was nearly 183 MHz, as shown in Figure 6c. When applying the additional pressure, ranging from 0 mmHg to 230 mmHg, the resonance frequency decreased from 183 MHz to 173 MHz, with a step (sensor’s sensitivity) of approximately 0.043 MHz/mmHg. The measured values of the resonance frequencies were lower than that of the target, it estimated to be uneven thicknesses of the inductor coil (as mentioned above) and a reduction in capacitance caused by the porosity of the capacitor. The reflow of AZ4620 photoresist is also assumed to contribute to this issue. This can be sufficiently improved by changes to the process parameters. Figure 6d shows that, by increasing the working distance between the sensor and external antenna, the resonance frequency curve of the phase was shifted to the low frequency region and gradually reduced the phase change. The resonance frequency of the pressure sensors used in the experiment was approximately 170 MHz. The different resonance behavior of the second sensor was due to the different values of the inductor and capacitor. The Q value of the sensor was ~10, which shows that the distance from the antenna is very closely related to the sensor’s sensitivity. Methods including additional antennae and the matching of the pressures sensor’s resonance frequency can be used to improve these distances by 10 mm.

**Figure 7:**
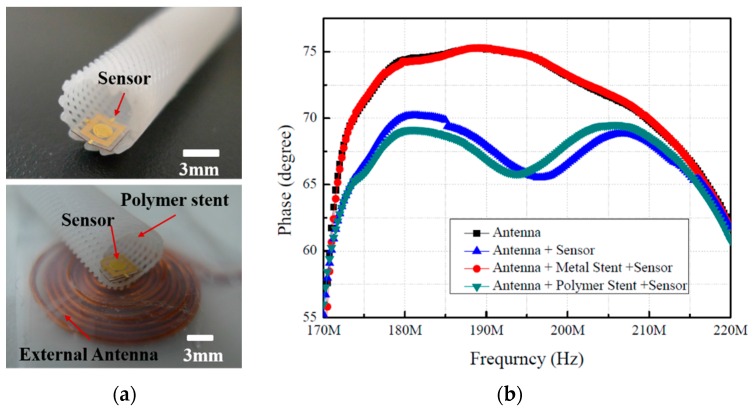
(**a**) Optical images of polymer stents integrated with wireless pressure sensors; (**b**) electrical influence of a metallic stent in the radio frequency (RF) experiments.

To evaluate the signal interference between the wireless pressure sensor and the metal or polymer stent, two types of stents were fabricated and tested. Moreover, a fundamental experiment was conducted regarding the applicability of the polymer stent inside an artificial tube identical to a blood vessel. The pressure sensor was integrated with the polymer stent using biodegradable glue, and RF tests were conducted, as shown in Figure 7. The plot of frequency against the phase for the interference between an external antenna and sensors is shown in Figure 7b. The negligible change in phase shift was observed in the case of the metal stent; hence, it exhibits much less sensitivity compared to the polymer stent. However, in the case of the polymer stent fabricated with the 3D printer, we confirmed that the change in resonance frequency was accurately followed by the pressure, owing to the nonexistence of interference between the sensor and the antenna. In this study, a conduit-type biodegradable polymer stent with a 5-mm diameter was used, and blood was substituted with water inside the tube to evaluate the artificial blood vessel. The effects of the interference between the polymer stent and the external antenna are shown in Figure 7b. These results indicate that a relatively high sensitivity value—similar to the one in the independent pressure sensor case—could be obtained in the case of the polymer stent, while at the same time experimentally confirming the excellency of the polymer stent’s RF compatibility. For future animal experiments, the design of the external antenna must be improved to enable the detection of the pressure changes. Improved versions of the external antenna and RF amplifier are expected to enable remote sensor operation at a distance of over 10 mm.

## 5. Conclusions

In conclusion, a polymer-based wireless micro pressure sensor was fabricated and evaluated. The measurement tests were performed after integrating the pressure sensor with a polymer stent fabricated with a 3D printer. The pressure sensor was fabricated using SU-8 polymer; an inductor coil and a MIM capacitor were integrated inside the pressure sensor for the wireless measurement of external pressure. The experiments were performed to determine the variation of resonance frequency as a function of change in pressure. When the pressure changed from 0 to 230 mmHg, the resonance frequency decreased from 183 MHz to 173 MHz, implying a measured sensor sensitivity of 0.043 MHz/mmHg. The change of capacitance resulting from the pressure change and the subsequent linear change of the sensor resonance frequency prove the sensor’s applicability as a biomedical wireless sensor. The signal interference between the sensor and the external antenna was conducted, and no interference was observed when the biodegradable polymer stent was used, which reflects an enormous improvement over the use of a metal stent. Monitoring of intravascular pressure using the polymer stent is therefore expected to be possible.
